# Integrating eye-tracking into psychometric research: computational approaches to explore response burden in questionnaire completion

**DOI:** 10.3389/fpsyg.2026.1757566

**Published:** 2026-06-24

**Authors:** Monica Casella, Francesca Borghesi, Elena Lupi, Pietro Cipresso, Davide Marocco

**Affiliations:** 1 Natural and Artificial Cognition Laboratory “Orazio Miglino”, Department of Humanistic Studies, University of Naples “Federico II”, Naples, Italy; 2 Department of Psychology, University of Turin, Turin, Italy

**Keywords:** eye-tracking, machine learning, psychometrics, response burden, self-reports

## Abstract

**Introduction:**

Long questionnaires can increase cognitive and motivational demands, causing response burden. This study investigates whether eye-movement behavior varies with instrument length.

**Methods:**

Twenty-five participants completed the BFI-44 and the longer BFQ-2 during gaze recording. Analysis included a 2 x 3 repeated-measures ANOVA (Test x Time) and the evaluation of seven machine-learning classifiers (including Random Forest and AdaBoost) trained on the final questionnaire segment.

**Results:**

Fixation, visit, glance, and pupil metrics decreased over time across both instruments. Significant Test x Time interactions revealed that the longer BFQ-2 produced more pronounced temporal variability in gaze behavior. The AdaBoost classifier achieved 73% accuracy in distinguishing between the two instruments.

**Discussion:**

Findings suggest that longer questionnaires elicit stronger temporal fluctuations in gaze, likely reflecting fatigue or strategic adaptation. Eye-tracking provides a valuable objective tool for assessing the stability of respondent engagement over time.

## 1 Introduction

Psychometric questionnaires are fundamental tools in psychological research, enabling the efficient and standardized collection of self-reported data on complex constructs such as personality, attitudes, and emotions. However, many widely used instruments are notably long. For example, popular personality measures like the NEO-PI-R (240 items), the MMPI-2-RF (338 items), and the California Psychological Inventory (434 items) often exceed 100 items (Bowling et al., 2021). While such length is often necessary to ensure construct validity and reliability, it raises important concerns about participant fatigue and data quality.

**Figure 1:**
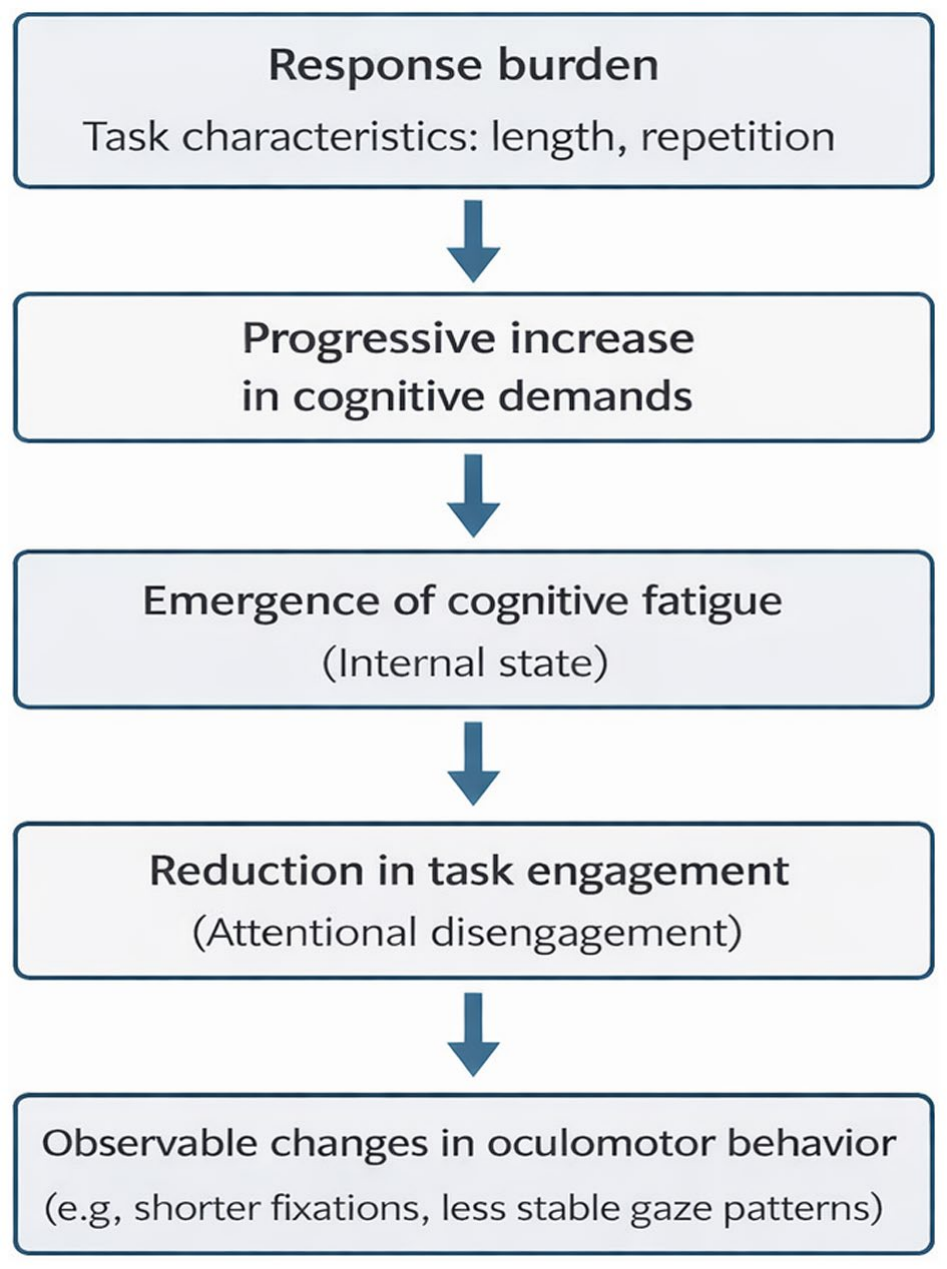
Conceptual model of response burden and oculomotor indicators. Response burden is conceptualized as a dynamic process linking task characteristics (e.g., length, repetition) to cognitive fatigue and reduced engagement, which are reflected in changes in oculomotor behavior such as shorter fixations and less stable gaze patterns.

These concerns are often framed within the broader concept of response burden, the cognitive and emotional effort required to complete a questionnaire or survey (Bradburn, 1977; Yan and Williams, 2022). Bradburn (1977) originally described burden as the interaction between task characteristics and the respondent’s perception, identifying duration, effort, frequency, and stress as its key components, the first three reflecting objective factors, and stress representing the subjective strain experienced by participants. Later models elaborated on this foundation: Haraldsen (2004) proposed that the subjective perception of burden mediates the impact of objective features such as questionnaire length on data quality, while Read (2019) distinguished between discrete and cumulative forms of burden, capturing how effort and fatigue evolve throughout completion. Yan et al. (2020) further integrated these perspectives, showing that low motivation, task difficulty, and negative perceptions jointly increase perceived burden, emphasizing its dynamic and multidimensional nature. Within this framework, it is important to distinguish response burden from related constructs such as cognitive fatigue and task engagement. Response burden refers to the cumulative demands imposed by the task (Bradburn, 1977; Yan and Williams, 2022), whereas cognitive fatigue represents an internal state arising from prolonged cognitive activity and resource depletion (Boksem and Tops, 2008; Hockey, 2013). Reduced engagement, in turn, reflects the observable behavioral manifestation of these processes, characterized by a decline in sustained attentional allocation and effort during task performance (Matthews et al., 2010; Hopstaken et al., 2015) (Figure 1).

Building on this theoretical background, recent frameworks conceptualize response burden as a fluctuating process that changes across the stages of survey participation, from initial expectations to accumulating cognitive load and eventual disengagement (Yan and Williams, 2022). Most existing research on response burden originates from survey methodology and performance testing, where it has traditionally been linked to cognitive load and time-on-task. In these contexts, burden is typically conceptualized as a problem of effortful processing: as instruments grow longer or more complex, they demand greater cognitive resources, leading to fatigue, slower responses, and higher measurement error. Yet, as noted by Yan and Williams (2022) and Giesen et al. (2011), in the case of self-report questionnaires, burden extends beyond cognitive strain to include motivational disengagement, boredom, and inattentive responding. In self-report contexts, response burden does not only reflect increased cognitive load, but also a progressive shift toward reduced engagement, where participants allocate fewer attentional resources to each item and rely on simplified response strategies. This pattern is commonly described in the survey methodology literature as satisficing behavior (Krosnick, 1991; Bowling et al., 2021).

Empirical evidence supports this progression. Bowling et al. (2021) found that participants tend to respond more carelessly as they progress through lengthy questionnaires, likely due to fatigue, boredom, or a sense that sufficient effort has already been invested. Experimental and meta-analytic studies further link longer questionnaires to higher dropout rates, reduced attention, and increased missing data (Rolstad et al., 2011; Yan et al., 2014).

Despite extensive research, the literature on response burden remains fragmented due to the diverse ways it has been operationalized, measured, and contextualized, ranging from performance-based tasks to self-report questionnaires assessing psychological constructs. For example, only a minority of studies have confirmed a clear link between questionnaire length and reduced response rates. Specifically, a meta-analysis found that just 6 of 25 empirical investigations reported lower participation for longer instruments, while the remaining studies offered no consistent evidence of such an effect (Rolstad et al., 2011). Consequently, the relationship between self-report questionnaire length, perceived burden, and its impact on response behavior remains uncertain. Most research on response burden has primarily focused on the processing of survey questions, examining indicators such as response time, item nonresponse, and rereading behavior as proxies of cognitive effort and difficulty. These approaches mainly capture how respondents comprehend and interpret items, rather than how they allocate attention during the response selection phase. However, in self-report questionnaires, particularly those using Likert-type scales, a substantial portion of the task involves selecting a response among predefined options. This response phase may be especially sensitive to reductions in engagement, as participants can rely on simplified or automatic strategies when cognitive effort declines. The present study extends existing approaches by focusing on oculomotor behavior during the response phase, providing a novel perspective on how response burden manifests not only in question processing but also in response selection dynamics.

To investigate this interaction, eye-tracking technology offers a promising methodological tool. Eye-tracking enables continuous, fine-grained measurement of visual attention and oculomotor behavior, and has been extensively used in applied performance research to evaluate mental workload, providing an objective window into the cognitive and affective processes underlying task performance. Importantly, eye-tracking metrics can reflect different underlying attentional processes depending on task characteristics. In cognitively demanding tasks, increases in fixation duration and pupil diameter are typically associated with greater cognitive effort and sustained attention (Zagermann et al., 2018). However, in repetitive and prolonged tasks such as self-report questionnaires, similar metrics may instead capture reductions in attentional engagement. In these contexts, shorter and less stable fixation patterns are more consistent with superficial processing and decreased cognitive investment rather than efficient performance (Hopstaken et al., 2015; Unsworth and Robison, 2016). Guo et al. (2021) examined the cognitive load of novice operators remotely controlling the Canadarm2 robotic arm under conditions of time pressure and latency. Variations in fixation duration and pupil dilation reliably reflected increased task difficulty, confirming that gaze-based indicators can sensitively track real-time fluctuations in workload. Similarly, in high-fidelity clinical simulations, pupil dilation has been shown to provide a measure of cognitive workload. Across numerous studies, larger pupil diameters and slower constriction rates have been associated with higher task demands, while fixation-based metrics distinguish novice from expert performance through differences in gaze efficiency and attentional focus (Wilbanks et al., 2021).

Evidence from visual computing and human-computer interaction research further supports these relationships. Zagermann et al. (2016, 2018) demonstrated that increases in task difficulty systematically produce longer fixation durations, higher saccade rates, and decreased blink frequency, an ensemble of markers reflecting rising cognitive load. They proposed a descriptive model linking voluntary (fixations, saccades) and involuntary (pupil dilation, blinks) eye movements to the three components of cognitive load described by Sweller (1988): intrinsic, extraneous, and germane. In this framework, fixation-related measures correspond to information processing and attention allocation, saccadic dynamics to visual search efficiency, pupil dilation to cognitive effort, and blink latency to attentional fatigue. These indicators together capture both the intensity and the temporal evolution of workload, providing a multidimensional perspective on mental effort. While these studies primarily interpret eye-tracking metrics in terms of cognitive load, their implications may differ in repetitive self-report contexts, where similar oculomotor patterns can reflect disengagement rather than increased effort.

In experimental psychology, Borys and Plechawska Wójcik (2017) applied these principles to compare visual attention patterns in experts and novices during perceptual and psychomotor tasks. Using the Ruff Figural Fluency Test, they found that participants with lower cognitive performance produced more fixations and revisited previous areas more frequently, suggesting higher cognitive effort and less efficient visual strategies.

In the domain of cognitive psychology applied to personality assessment, Berkovsky et al. (2019) investigated whether eye-tracking metrics “such as fixations, saccades, movement velocity, and amplitude” could predict Big Five personality traits (Openness, Conscientiousness, Extraversion, Agreeableness, and Neuroticism). By exposing participants to various visual stimuli, the study demonstrated that these oculometric measures effectively predicted personality traits.

In applied psychology contexts, Millecamp et al. (2021) explored the classification of personal characteristics, including Big Five Openness and Need for Cognition, during user interactions with a music recommendation system interface. Gaze patterns were recorded as participants navigated the system and used to train automated classifiers. The findings revealed that oculometric patterns vary with cognitive load, task complexity, and information processing demands.

Taken together, these studies demonstrate that eye-tracking provides a robust and non, intrusive means of quantifying cognitive load and attentional dynamics during complex tasks.

However, eye-tracking indicators can reflect two distinct yet interrelated processes: cognitive effort and attentional disengagement. In performance contexts, increased task difficulty typically leads to longer fixations and greater pupil dilation, reflecting intensified cognitive processing (Zagermann et al., 2018; Borys and Plechawska Wójcik, 2017). In contrast, in prolonged and repetitive tasks such as Likert-type questionnaires, changes in the same metrics may signal a progressive withdrawal of attention and effort. In these cases, shorter fixation durations, reduced fixation counts, and decreased pupil size are more consistent with attentional disengagement and reduced engagement, rather than improved processing efficiency. This pattern emerged in the preliminary study by Casella et al. (2026), where participants completed two personality questionnaires of different lengths, the Big Five Inventory (BFI-44) and the Big Five Questionnaire (BFQ-2, 134 items), while their eye movements were recorded. Despite a limited sample (*N* = 5), the findings showed shorter fixation durations, fewer fixations and saccades, and smaller pupil diameters during the longer questionnaire, suggesting a progressive attentional disengagement associated with cumulative response burden.

Building on this foundation, the present study combines inferential statistics and machine learning analyses to capture both univariate and multivariate patterns in eye-movement behavior. Inferential analyses provide an initial metric-by-metric examination of how gaze features vary across questionnaire sections and between tests of different lengths. However, because response burden is inherently multidimensional, relying solely on single-metric tests may overlook complex dependencies among fixation-, visit-, glance-, and pupil-based measures. For this reason, we complement inferential analyses with supervised machine-learning classifiers, which evaluate all gaze metrics jointly and assess whether patterns of ocular behavior can reliably distinguish between short and long questionnaires. Furthermore, we integrate this multivariate approach with post-hoc model explainability, using permutation-based feature importance to determine which gaze metrics, when considered within the full feature space, contribute most to predictive performance. This approach is particularly suitable in this context, as response burden is expected to emerge as a distributed pattern of changes across multiple oculomotor indicators rather than as isolated effects on single metrics. This study contributes to bridging the gap between survey methodology and cognitive models of attention by providing objective behavioral markers of response burden.

## 2 Materials and research methods

### 2.1 Research design

This study represents a significant methodological extension of the exploratory work by Casella et al. (2026). While the preliminary work suggested that longer questionnaires elicit fewer and shorter fixations toward the end, it was restricted to a small descriptive sample (*N* = 5) and only compared the initial and final task segments. The present work expands this framework by: (1) increasing the sample to 25 participants to allow for inferential testing; (2) adding a midpoint analysis to provide a more continuous view of how visual behavior evolves across the task.; and (3) employing supervised machine learning classifiers and permutation, based explainability to identify the specific multivariate signatures that distinguish ocular behavior in short versus long instruments.

The participants complete two personality questionnaires of different lengths: the Big Five Inventory (BFI-44, 44 items) and the Big Five Questionnaire (BFQ-2, 134 items). Both instruments have been validated for the Italian population, ensuring linguistic and cultural equivalence.

The study adopts a within-subject experimental design, allowing each participant to complete both questionnaires 1 week apart. This structure enables direct comparison of ocular and behavioral responses associated with short versus long self-report instruments while controlling for individual differences in visual and attentional patterns.

### 2.2 Participants

The final sample comprised 25 undergraduate Psychology students from the University of Naples Federico II, aged between 18 and 30 years (see Table 1 for demographic characteristics). Two partially missing observations were identified, involving Subject 7 in the Initial condition and Subject 23 in the Middle condition. This age range was chosen because it typically ensures good overall health, normal visual acuity, and greater scheduling flexibility, thereby facilitating consistent and reliable participation in the study.

**Table 1:** Demographic characteristics of participants.

Category	Subcategory	Value
Age	Mean (years)	21.84
Gender	Female	18
	Male	7
Education	Graduates	24
	Master’s degree (male)	1
Dominant hand	Right-handed	18
	Left-handed	5
	Left-handed but used right hand	3 of 5 left, handed

Based on the G*Power analysis we ran, we estimated the sample size required for a repeated-measures ANOVA with six within-subject measurements, based on Time (Initial, Middle, End) * Test (BFI-44 vs. BFQ-2). We specified a medium effect size (*f* = 0.30), an alpha level of 0.05, and a desired statistical power of 0.95. We also assumed a moderate correlation among repeated measures (*r* = 0.40) and perfect sphericity (*ε* = 1).

Under these conditions, G*Power indicates that 24 participants are required to achieve the desired level of power. The analysis yields a noncentrality parameter *λ* of 21.60, a critical *F* value of 2.29, 5 numerator degrees of freedom, and 115 denominator degrees of freedom.

All procedures were conducted in accordance with the Ethical Committee of Psychological Research of the Department of Humanities at the University of Naples Federico II, which reviewed the study project and attested its conformity to ethical norms (protocol n° 2/2024, approved on 29/02/2024).

### 2.3 Instruments

#### 2.3.1 Big five inventory (BFI-44)

The BFI-44 (John et al., 1991) is a concise measure of the Big Five personality traits: openness, conscientiousness, extraversion, agreeableness, and neuroticism. It consists of 44 items rated on a five-point Likert scale (1 = Strongly disagree to 5 = Strongly agree). The instrument has been extensively validated.

including in Italian samples (Ubbiali et al., 2013), and is widely used in research where efficiency and participant engagement are priorities.

#### 2.3.2 Big five questionnaire (BFQ-2)

The BFQ-2 (Caprara et al., 2008) is a measure of the same five personality dimensions, comprising 134 items rated on a five-point Likert scale (1 = Very false for me to 5 = Very true for me). Each factor includes two facets:

- Extraversion: Dynamism, Dominance
- Agreeableness: Empathy, Politeness
- Conscientiousness: Scrupulousness, Perseverance
- Emotional Stability: Emotional Control, Impulse Control
- Openness: Openness to Culture, Openness to Experience

The BFQ-2 also includes a Lie (LIE) scale to control for social desirability. It has been validated in Italian population and is considered a comprehensive instrument for personality assessment.

### 2.4 Procedure

After providing informed consent, participants were tested individually in a quiet, controlled environment with stable lighting and minimal distractions. The Tobii Pro Nano eye-tracker was calibrated for each participant to ensure accurate gaze recording.

Participants completed both the Big Five Inventory (BFI-44) and the Big Five Questionnaire (BFQ-2) via the Qualtrics online platform. To maximize ecological validity, we employed a single-page, scrollable layout for all questionnaire items, thereby avoiding the artificial constraints typically associated with one-item-per-page designs. This configuration encouraged naturalistic interaction with the instruments, reflecting the fluid navigation common in real-world digital assessments. To ensure the reliability of recordings across participants and sessions, all trials were conducted in a quiet, controlled environment with standardized display parameters (including constant screen luminance, dimensions, and resolution) and stable ambient lighting.

Each questionnaire was completed in a separate session 1 week apart, minimizing learning and carryover effects while maintaining consistent testing conditions. The order of questionnaire administration was counterbalanced across participants using an alternating assignment procedure. Specifically, participants were assigned sequentially to one of the two possible administration orders: the first participant completed the BFI first and the BFQ in the subsequent session, the second participant completed the BFQ first and the BFI in the subsequent session, and so on.

**Figure 2:**
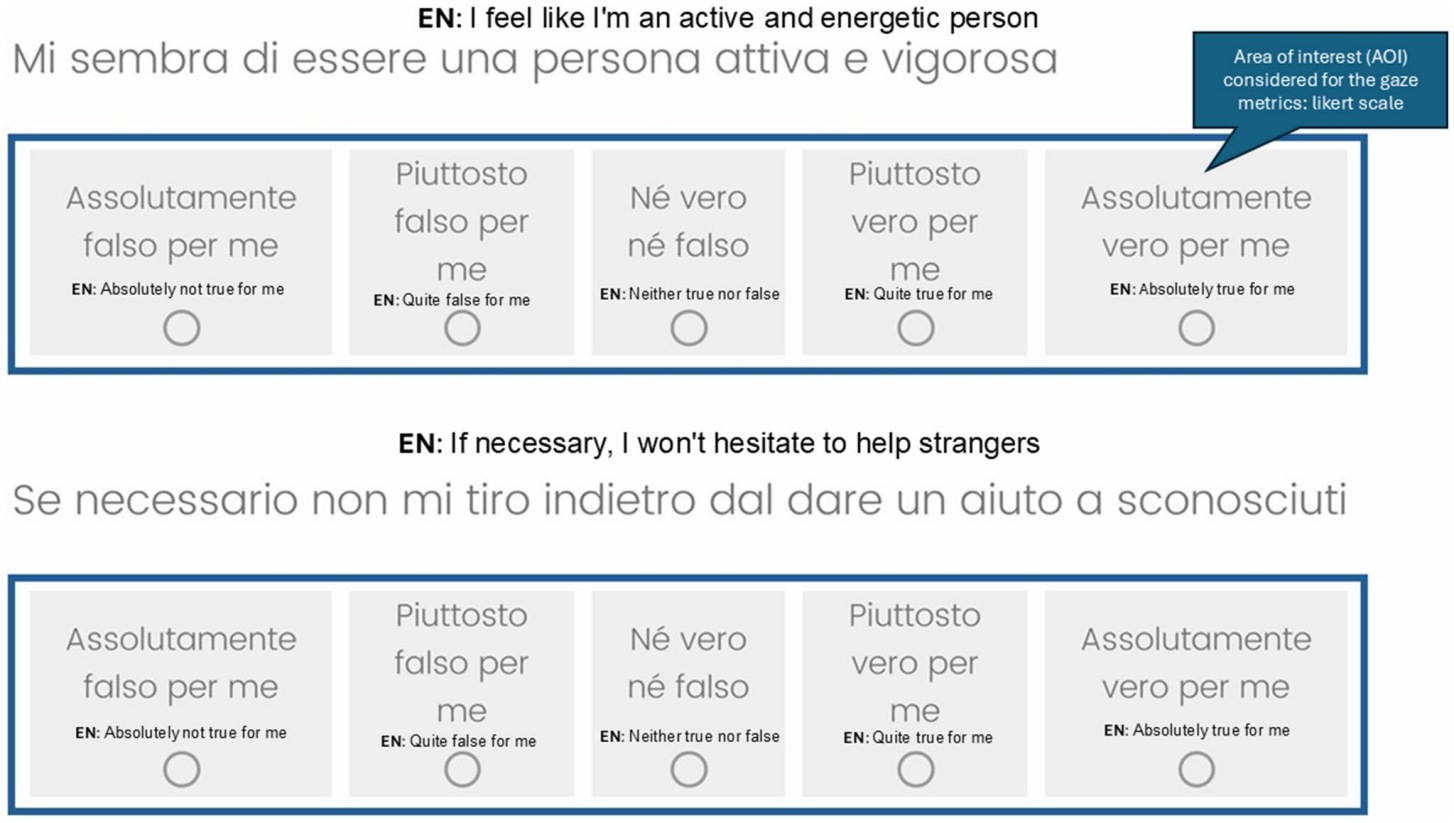
Examples of area of interests (AOI) defined over the full Likert-scale response section. The AOI included the entire response bar, considering all the five options.

To investigate the temporal evolution of potential response burden, we extracted eye-tracking data for specific item clusters within each questionnaire. We focused on the first five items, a midpoint segment (items 21–25 for the BFI-44 and items 63–67 for the BFQ-2), and the final five items. This 5-item window was selected as a methodological compromise to ensure metric stability while preserving sensitivity to temporal shifts. Single-item gaze metrics are often susceptible to high intra-individual variability; thus, aggregating over a small cluster helps stabilize the signal against idiosyncratic outliers without losing the fine-grained temporal resolution required to identify transitions in engagement or time-on-task effects. Furthermore, using a fixed-size window across both the BFI-44 and BFQ-2 facilitated a direct comparison of visual behavior at comparable task stages, independent of total instrument length. Importantly, items text was not used as an Area of Interest (AOI) because they differ considerably in length and linguistic complexity, which would introduce unwanted variability and compromise the interpretability of gaze metrics. Instead, the entire Likert response bar for each item was treated as a single, unified AOI. This approach allowed us to capture the visual behavior related to the response selection across the full scale, while specifically excluding the item text. (Figure 2).

This approach allowed us to examine visual attention and the decision, making processes associated with response selection, rather than reading or comprehension alone, while also capturing changes in attentional engagement as participants progressed through the questionnaire.

### 2.5 Eye-related metrics

For each questionnaire item, a set of fixation-, visit-, glance-, and pupil- based metrics was extracted from the raw gaze signal.

#### 2.5.1 Fixation metrics

- Total Duration of Fixations: Sum of all fixation durations (ms) within an Area of Interest (AOI), indexing the overall visual processing allocated to the item.
- Average Fixation Duration: Mean duration of fixations (ms), commonly interpreted as an indicator of cognitive load (longer fixations reflect increased processing effort).
- Minimum / Maximum Fixation Duration: Lower and upper bounds of fixation duration within the AOI (ms).
- Number of Fixations: Total fixation count within the AOI, reflecting how frequently attention was directed to the item.

#### 2.5.2 Pupil metric

- Average Pupil Diameter: Mean pupil size (mm) during AOI exposure. Tobii Pro Nano provides binocular pupil-diameter recordings, and averages were computed across valid samples.

#### 2.5.3 Visit metrics

A visit represents a continuous sequence of gaze samples within the same AOI, regardless of whether it contains fixations.

- Total Visit Duration: Cumulative duration of all visits to an AOI (ms).
- Average Visit Duration: Mean duration of individual visits, reflecting sustained engagement with the item (ms).
- Minimum / Maximum Visit Duration: Shortest and longest uninterrupted visits (ms).
- Number of Visits: Count of discrete visits, indexing gaze returns that may reflect re, reading or verification behavior.

#### 2.5.4 Glance metrics

Glances correspond to short gaze engagements in an AOI typically capturing rapid checks distinct from longer visits.

- Total Glance Duration: Sum of all glance durations.
- Average Glance Duration: Mean duration of glances, associated with quick scanning.
- Minimum/Maximum Glance Duration: Shortest and longest glances recorded.
- Number of Glances: Total count of glance events.

### 2.6 Inferential univariate analysis

The study used a fully within-subjects 2 × 3 design, crossing the factor Test (BFQ-2 vs. BFI-44) with the factor Time (e.g., items’ position in terms of Initial, Middle, End). All participants completed every condition, yielding repeated measurements across both dimensions. Although the Time factor was defined within each questionnaire (i.e., nested within Test at the item level), the analyses were conducted on aggregated participant, level metrics rather than individual item responses. Specifically, eye-tracking measures were averaged within each Test × Time condition, resulting in six observations per participant. This approach model the data using a repeated-measures ANOVA with Test and Time as within-subject factors, focusing on overall patterns of interaction with the task rather than item-level variability. For the Time factor with three levels, violations of the sphericity assumption were assessed and, where necessary, Greenhouse, Geisser corrections were applied. To control for Type I error inflation due to multiple dependent variables, we applied a Bonferroni correction across the 13 eye-movement measures included in the analyses (spanning Visits, Glances, Fixations, and Pupil Diameter). Accordingly, the adjusted significance threshold was set to *α* = 0.004 (0.05/13), reflecting a conservative control of family-wise error across the full set of tested outcomes. For *post hoc* comparisons, we applied Tukey’s honestly significant difference (HSD) correction to account for multiple pairwise tests while maintaining statistical power.

### 2.7 Machine learning

#### 2.7.1 Selected algorithms and learning parameters

The selected algorithms cover a broad spectrum of learning paradigms, linear, kernel, based, and ensemble methods, providing complementary strengths for handling behavioral data characterized by complex patterns.

Logistic Regression (Menard, 2001) was included as a linear probabilistic baseline. By modeling the log-odds of the target as a linear combination of features, it offers a transparent decision function and has previously been used in gaze, classification and oculomotor, behavior studies (e.g., Cybulski, 2025; Ma and Jia, 2024).

To incorporate margin-based learning, we employed a Linear Support Vector Machine (SVM), which identifies the hyperplane that maximizes class separation under a hinge-loss objective (Tang, 2013). Although also linear, its optimization criterion differs substantially from logistic regression, often yielding better generalization when classes are not easily separable. Because eye-movement metrics frequently exhibit nonlinear relationships, we further included an RBF-kernel SVM, which maps the input space into a higher-dimensional feature space via a Gaussian kernel (Amari and Wu, 1999). This makes the model suited to capturing complex decision boundaries commonly observed in gaze-based behavioral data (e.g., Zheng et al., 2020; Pritalia et al., 2020).

For ensemble learning, four tree-based models were examined. Random Forests (Breiman, 2001) aggregate multiple bootstrapped trees built on randomly sampled feature subsets, offering robustness to multicollinearity and noisy measurements, frequent characteristics of eye-tracking variables. Extremely Randomized Trees (Extra Trees; Geurts et al., 2006) introduce additional stochasticity by selecting split thresholds at random, typically reducing variance and computational cost while maintaining strong predictive stability. Such properties are beneficial when dealing with heterogeneous oculomotor measures that may interact in unpredictable ways (e.g., Mao et al., 2020; Ni et al., 2025).

AdaBoost (Schapire, 2013) was included as a sequential boosting method that iteratively up, weights misclassified samples. Its emphasis on hard-to-separate cases can highlight subtle discriminative patterns in eye-movement behavior, and it has been applied in prior gaze-analysis applications (e.g., Ma and Jia, 2024). Gradient Boosting Machines (Natekin and Knoll, 2013) extend this logic by fitting successive weak learners to the negative gradient of a differentiable loss function. Their flexibility in regularization, learning,-rate control, and subsampling makes them well-suited for capturing fine, grained structure in psychophysiological data, including eye-tracker measures (e.g., Salima et al., 2023).

All models were implemented within a two-stage pipeline. In the benchmarking phase, each classifier was evaluated using its default configuration. Subsequently, the best-performing nonlinear models, Random Forest, Extra Trees, Gradient Boosting, and RBF-SVM, were refined through randomized hyperparameter search (25 configurations per model). Optimization was performed using a stratified 3-fold cross-validation scheme, with mean cross-validated accuracy as the selection criterion.

#### 2.7.2 Explainability analysis

To enhance the interpretability of the final model, we performed a *post hoc* explainability analysis.

To quantify the contribution of each gaze feature to the final classifier’s performance, we applied permutation importance on the held-out test set implemented in scikit-learn (Pedregosa et al., 2011). Permutation importance estimates how much a model’s predictive accuracy deteriorates when the association between a single feature and the outcome is disrupted, while all other features and the data distribution are kept intact. This model-agnostic method is widely used in interpretability research due to its simplicity and effectiveness, particularly for complex, high-dimensional models (Altmann et al., 2010).

After selecting the best model through cross-validation and hyperparameter tuning, we refit it on the training split and evaluated feature importance exclusively on the test set. For each feature in turn, we randomly permuted its values across test instances (thereby breaking any learned relationship between that feature and the label but preserving the joint structure of all remaining variables), recomputed the model’s score, and recorded the drop in test accuracy relative to the unpermuted baseline. This process was repeated 200 times per feature to average over sampling variability.

The importance of a feature is defined as the mean decrease in accuracy across repetitions; larger positive decreases indicate greater importance. For reporting, we ranked features by their mean decrease and focused on the top-10 contributors.

Permutation importance is model-agnostic and directly tied to out-of-sample performance, making it well suited to evaluate how much each variable contributes to the model’s generalization on new data. It has been shown to be particularly useful in domains requiring high interpretability, such as several health domains (e.g., Gómez et al., 2020; Mi et al., 2021). A larger mean drop in accuracy for a given feature implies that the classifier depends more strongly on that signal to produce correct predictions. Near-zero (or negative) mean drops suggest weak or redundant contributions under the current feature set and model.

## 3 Results

### 3.1 Inferential results

Eye-movement metrics were organized into four macro-categories: Fixation measures (e.g., total, average, minimum, and maximum fixation duration, and number of fixations), Glance measures (e.g., total, average, minimum, and maximum duration), Pupil measures, and Visit measures (e.g., total, average, minimum, and maximum duration). Detailed descriptive statistics for all eye-movement metrics across conditions are presented in the Supplementary Tables 2–5.

To evaluate differences in eye-movement patterns across test conditions, a series of repeated-measures ANOVAs was conducted on all eye-movement metrics, using Test (BFQ-2 vs. BFI-44) and Time (Initial, Middle, End) as within-subject factors.

#### 3.1.1 Fixation measures

For the average number of fixations, there was a significant main effect of Time, *F*(2, 44) = 9.22, *p* < 0.001, η^2^ₚ = 0.295, and a significant Test × Time interaction, *F*(2, 44) = 11.90, *p* < 0.001, η^2^ₚ = 0.351. The main effect of Test was not significant, *F*(1, 22) = 0.02, *p* = 0.887.

For the minimum fixation duration, significant main effects emerged for Time, *F*(2, 44) = 11.94, *p* < 0.001, η^2^ₚ = 0.352, and for Test, *F*(1, 22) = 4.65, *p* = 0.042, though this effect does not meet the corrected *α* = 0.004 threshold. The Test × Time interaction was significant, *F*(2, 44) = 9.17, *p* < 0.001, η^2^ₚ = 0.294.

For the maximum fixation duration, the main effect of Time was significant, *F*(2, 44) = 14.62, *p* < 0.001, η^2^ₚ = 0.399. The main effect of Test was not significant, *F*(1, 22) = 0.92, *p* = 0.348, and the Test × Time interaction did not reach the corrected significance criterion, *F*(2, 44) = 4.63, *p* = 0.015.

For the total duration of fixations, only the main effect of Time was significant, *F*(2, 44) = 46.39, *p* < 0.001, η^2^ₚ = 0.678. Neither the main effect of Test, *F*(1, 22) = 0.11, *p* = 0.747, nor the interaction, *F*(2, 44) = 0.14, *p* = 0.869, were significant.

**Figure 3:**
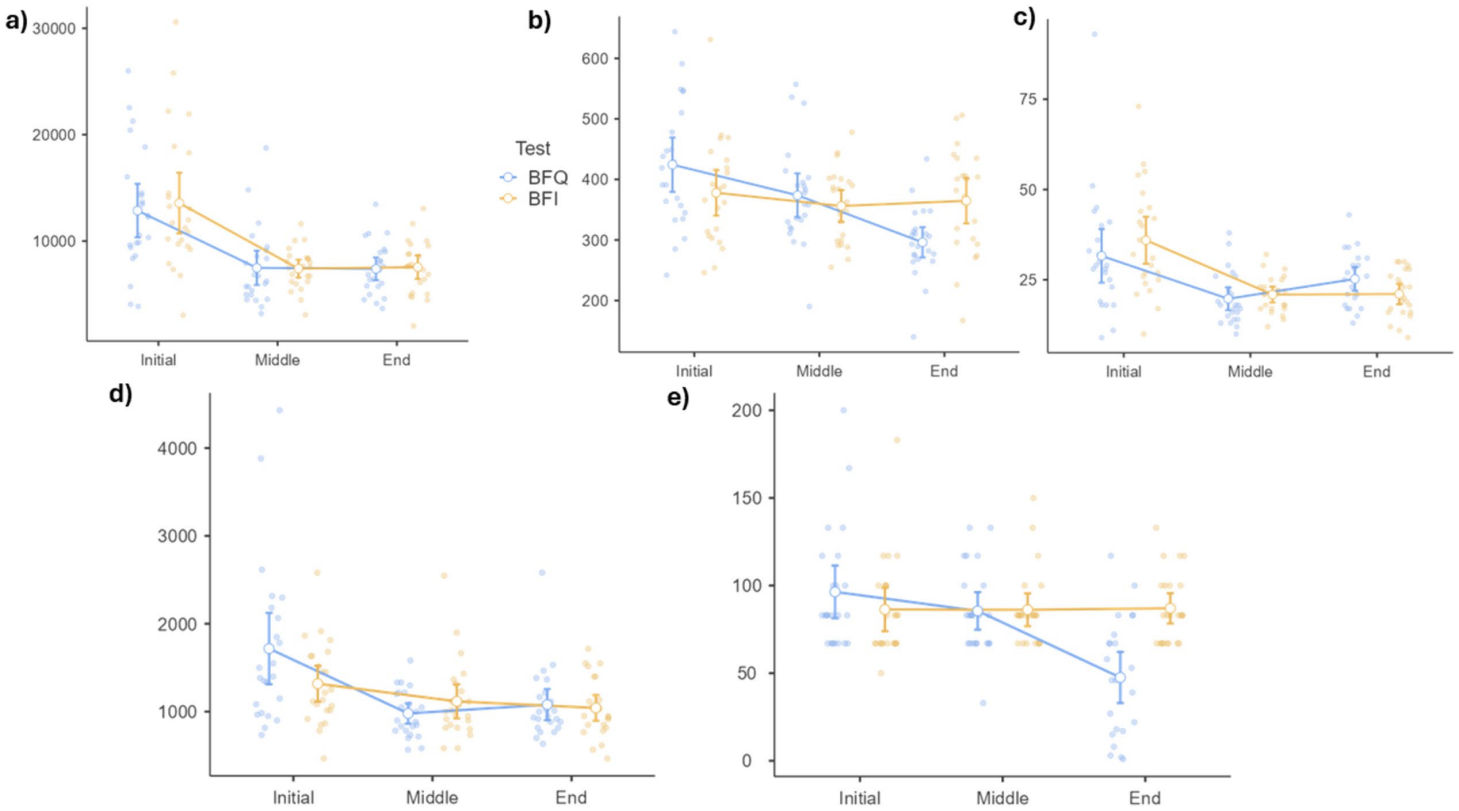
Fixations results divided for **(a)** total fixation, **(b)** average durations of fixations, **(c)** number of fixations, **(d)** maximum fixations, **(e)** minimum fixations.

The number of fixations showed a significant main effect of Time, *F*(2, 44) = 17.70, *p* < 0.001, η^2^ₚ = 0.446. Neither the main effect of Test, *F*(1, 22) = 0.07, *p* = 0.794, nor the Test × Time interaction, *F*(2, 44) = 2.41, *p* = 0.101, were significant at *α* = 0.01. Figure 3 shows all the results about fixations.

#### 3.1.2 Visit measures

For the number of visits, the main effect of Time was significant, *F*(2, 44) = 13.99, *p* < 0.001, η^2^ₚ = 0.389, and the Test × Time interaction was not significant, *F*(2, 44) = 4.65, *p* = 0.015, since this does not reach α = 0.004. The main effect of Test was not significant, *F*(1, 22) = 0.55, *p* = 0.465.

For the total duration of visits, only the main effect of Time was significant, *F*(2, 44) = 41.66, *p* < 0.001, η^2^ₚ = 0.654. The main effect of Test, *F*(1, 22) = 0.19, *p* = 0.664, and the interaction, *F*(2, 44) = 0.35, *p* = 0.708, were not significant.

For the average visit duration, the main effect of Time was significant, *F*(2, 44) = 38.21, *p* < 0.001, η^2^ₚ = 0.635. Neither the main effect of Test, *F*(1, 22) = 3.78, *p* = 0.065, nor the interaction, *F*(2, 44) = 0.88, *p* = 0.421, reached significance at *α* = 0.01.

For the maximum visit duration, the main effect of Time was significant, *F*(2, 44) = 29.70, *p* < 0.001, η^2^ₚ = 0.575, whereas the main effect of Test, *F*(1, 22) = 0.59, *p* = 0.452, and the interaction, *F*(2, 44) = 1.20, *p* = 0.312, were not significant.

**Figure 4:**
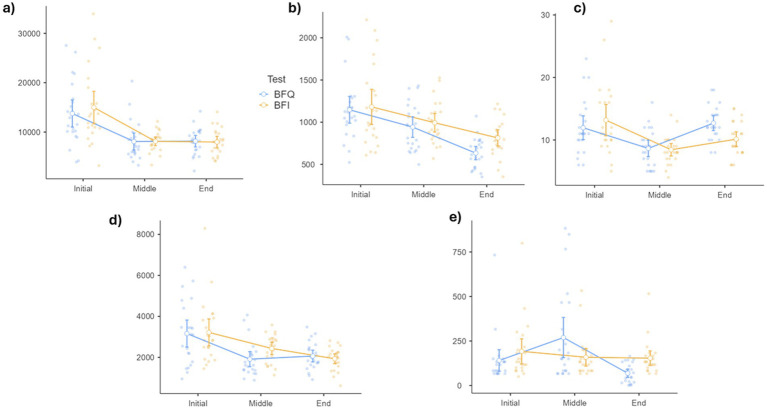
Visit measures divided by **(a)** total duration of visit, **(b)** average duration of visit, **(c)** number of visit, **(d)** maximum duration of visit, **(e)** minimum duration of visit.

For the minimum duration of visits, the main effect of Time, *F*(2, 44) = 5.54, *p* = 0.007, η^2^ₚ = 0.201, and the Test × Time interaction, *F*(2, 44) = 5.24, *p* = 0.009, η^2^ₚ = 0.192, did not reach significance after applying the Bonferroni, corrected threshold (α = 0.004). The main effect of Test was not significant, *F*(1, 22) = 0.12, *p* = 0.73. Figure 4 shows all results for visit measures.

#### 3.1.3 Glance measures

For the total duration of glances, the main effect of Time was significant, *F*(2, 44) = 41.44, *p* < 0.001, η^2^ₚ = 0.653. Neither the main effect of Test, *F*(1, 22) = 0.33, *p* = 0.570, nor the Test × Time interaction, *F*(2, 44) = 0.30, *p* = 0.743, were significant.

For the average glance duration, the main effect of Time was significant, *F*(2, 44) = 39.49, *p* < 0.001, η^2^ₚ = 0.642. The main effect of Test was not significant after correction for multiple comparisons, *F*(1, 22) = 4.28, *p* = 0.050, and the interaction was not significant, *F*(2, 44) = 1.04, *p* = 0.363.

For the number of glances, the main effect of Time was significant, *F*(2, 44) = 13.99, *p* < 0.001, η^2^ₚ = 0.389. The Test × Time interaction was significant at the uncorrected level, *F*(2, 44) = 4.65, *p* = 0.015, but did not remain significant after correction for multiple comparisons (*α* = 0.004). The main effect of Test was not significant, *F*(1, 22) = 0.55, *p* = 0.465.

For the maximum glance duration, the main effect of Time was significant, *F*(2, 44) = 29.68, *p* < 0.001, η^2^ₚ = 0.574. Neither the main effect of Test, *F*(1, 22) = 0.59, *p* = 0.449, nor the interaction, *F*(2, 44) = 1.15, *p* = 0.327, were significant.

**Figure 5:**
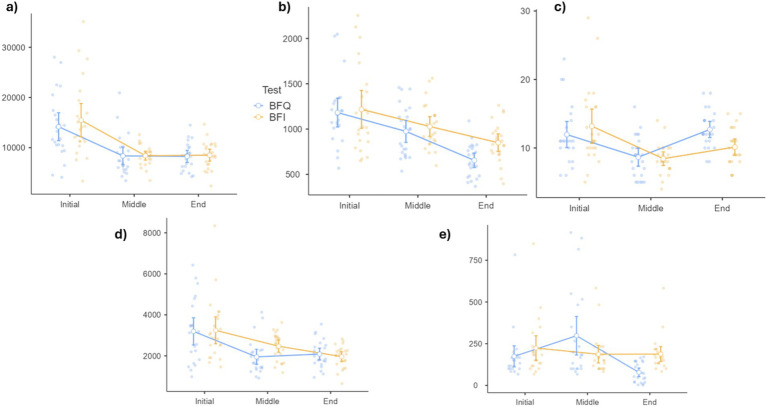
Glances measures divided by **(a)** total duration of glances, **(b)** average, **(c)** number, **(d)** maximum, **(e)** minimum.

For the minimum glance duration, the main effect of Time, *F*(2, 44) = 5.79, *p* = 0.006, η^2^ₚ = 0.208, and the Test × Time interaction, F(2, 44) = 5.70, *p* = 0.006, η^2^ₚ = 0.206, were significant at the uncorrected level but did not survive correction for multiple comparisons (α = 0.004), although both effects were close to the adjusted threshold. The main effect of Test was not significant, *F*(1, 22) = 0.43, *p* = 0.517. Figure 5 shows all results about glances.

#### 3.1.4 Pupil measures

**Figure 6:**
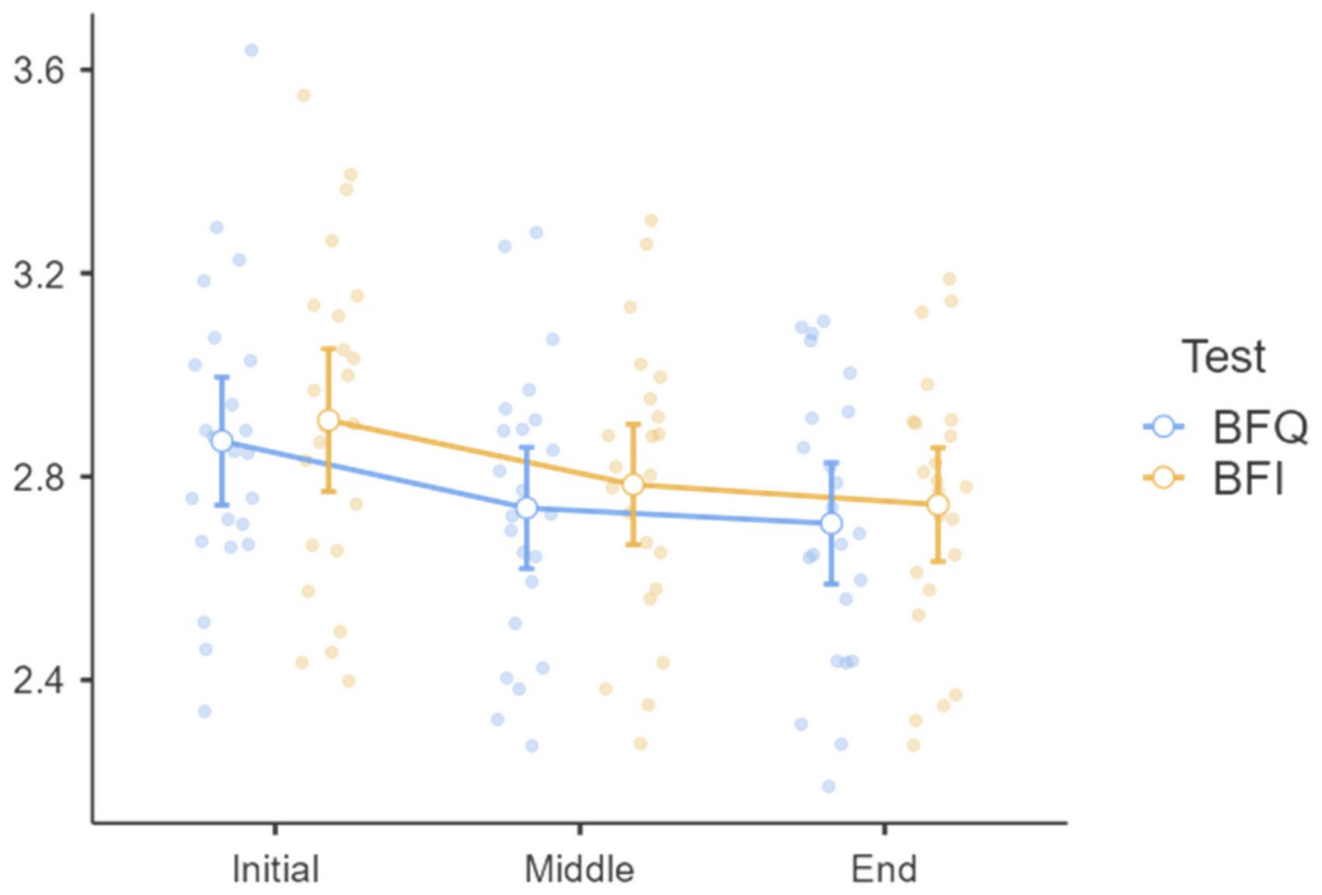
Average pupil diameter.

For average pupil diameter, the main effect of Time was significant, *F*(2, 44) = 40.57, *p* < 0.001, η^2^ₚ = 0.648. Neither the main effect of Test, *F*(1, 22) = 0.57, *p* = 0.457, nor the Test × Time interaction, *F*(2, 44) = 0.09, *p* = 0.911, were significant (Figure 6).

### 3.2 Machine learning analysis

Given that the inferential analyses revealed a significant effect of time on several oculomotor indicators, suggesting that gaze behavior changed as participants progressed through the questionnaires, we narrowed the focus on the last five items response categories, where these temporal patterns were most evident. In particular, including initial and middle segments would have likely led to suboptimal model learning due to the lack of discriminative variance in the early feature space. We therefore targeted the final segment as a “time-sensitive window” where the cumulative effects of questionnaire length were expected to be most manifest. To assess whether the two questionnaires (BFI-44 and BFQ-2) could be distinguished solely through oculomotor behavior in this time-sensitive segment, we complemented the univariate inferential findings with a multivariate perspective by training seven supervised machine learning classifiers using the two questionnaires as class labels.

#### 3.2.1 Algorithms performance evaluation

The set of baseline classifiers included: logistic regression, linear support vector machine (SVM with a linear kernel), SVM with a radial basis function kernel (RBF-SVM), random forest, extremely randomized trees, AdaBoost, and gradient boosting. Performance was quantified using stratified three-fold cross-validation and models were then ranked according to mean cross-validated accuracy.

Following the baseline comparison reported in Table 2, we conducted hyperparameter optimization on the four strongest contenders.

**Table 2:** Performance metrics for all evaluated models, reported as the average scores across the three cross-validation folds.

Model	Accuracy	Precision	Recall	F1
AdaBoost	0.728	0.754	0.671	0.706
GradBoost	0.700	0.710	0.671	0.689
LogReg	0.692	0.697	0.671	0.682
RandomForest	0.656	0.657	0.648	0.652
Linear-SVM	0.656	0.659	0.639	0.646
RBF-SVM	0.652	0.649	0.672	0.659
ExtraTrees	0.644	0.659	0.600	0.628

We use randomized search with 25 iterations per model. Each candidate configuration was evaluated with a stratified three-fold cross-validation, using mean accuracy as the selection criterion. The hyperparameter ranges explored for each algorithm are included in the Supplementary Table 1. After the randomized hyperparameter search, the AdaBoost classifier emerged as the best-performing model, achieving the highest cross-validated accuracy (73%) under this feature set. The final performance metrics were then computed using a single train-test split, with 30% of the data reserved for testing, and the resulting test-set outcomes are reported in Table 3.

**Table 3:** Classification performance metrics for the selected AdaBoost model, computed on the test-set (30%).

**Class**	**Precision**	**Recall**	**F1-score**	**Support**
BFI-44	0.683	0.800	0.737	35
BFQ-2	0.794	0.675	0.730	40
Accuracy			0.733	75

AdaBoost showed balanced performance across the two questionnaires, with higher recall for BFI-44 items and higher precision for BFQ-2 items. Specifically, the model correctly identified BFI-44 responses with a recall of 0.800 and an F1-score of 0.737, whereas BFQ-2 responses were recognized with a precision of 0.794 and an F1-score of 0.730. Overall accuracy on the test set was 0.733 (*N* = 75), indicating that the classifier generalized well beyond the training sample. These results highlight that AdaBoost captured discriminative gaze-based patterns associated with each questionnaire type while maintaining a stable balance between false positives and false negatives.

**Figure 7:**
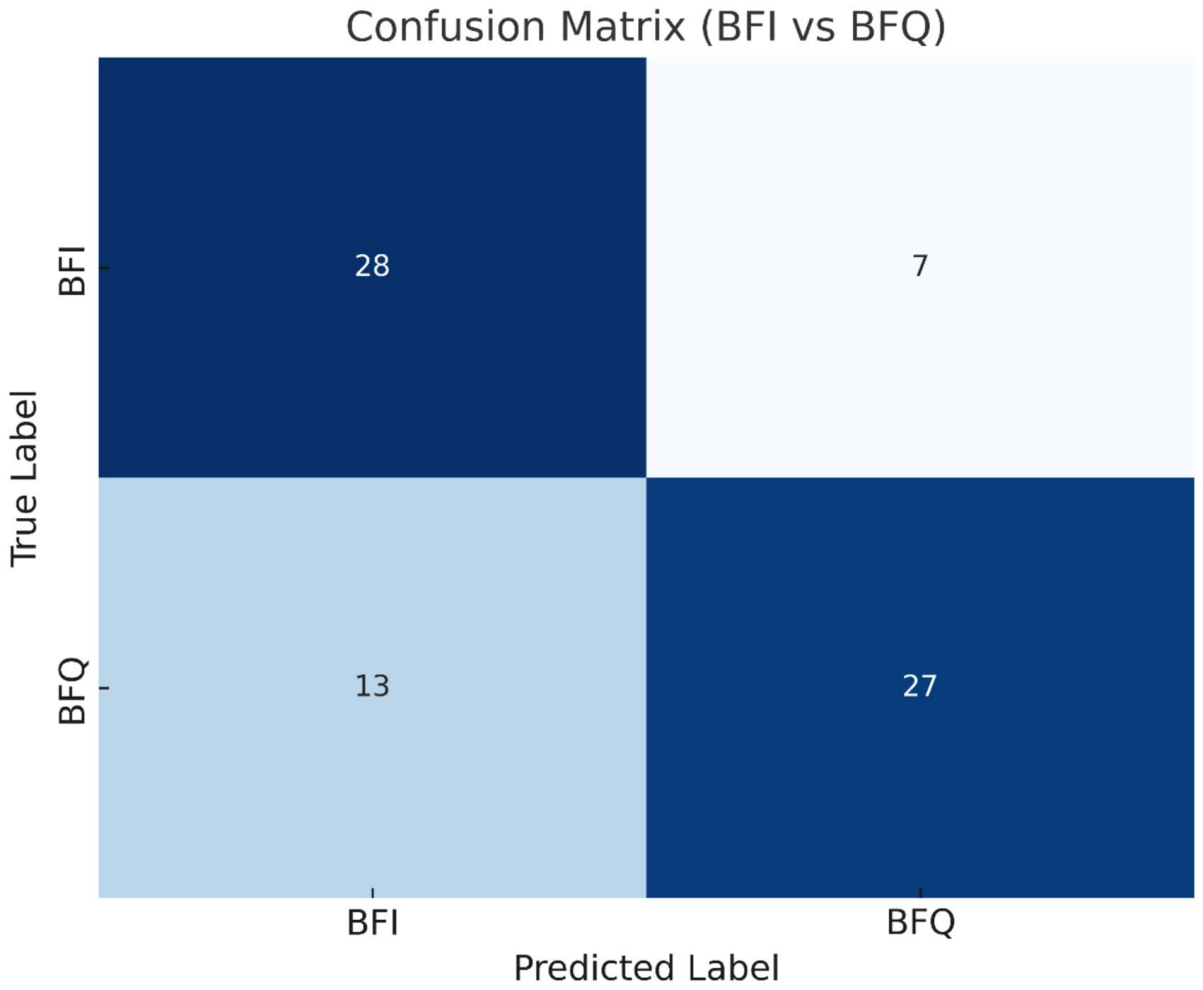
Confusion matrix for the AdaBoost classification of questionnaire type (BFI-44 vs. BFQ-2) on the test set.

According to the confusion matrix (Figure 7), misclassifications were relatively balanced, with 7 BFI-44 items incorrectly assigned to the BFQ-2 class and 13 BFQ-2 items predicted as BFI-44. This asymmetry reflects the pattern already observed in the precision-recall scores: the model produces fewer false positives for this class but a higher number of false negatives.

#### 3.2.2 Explainability analysis

**Figure 8:**
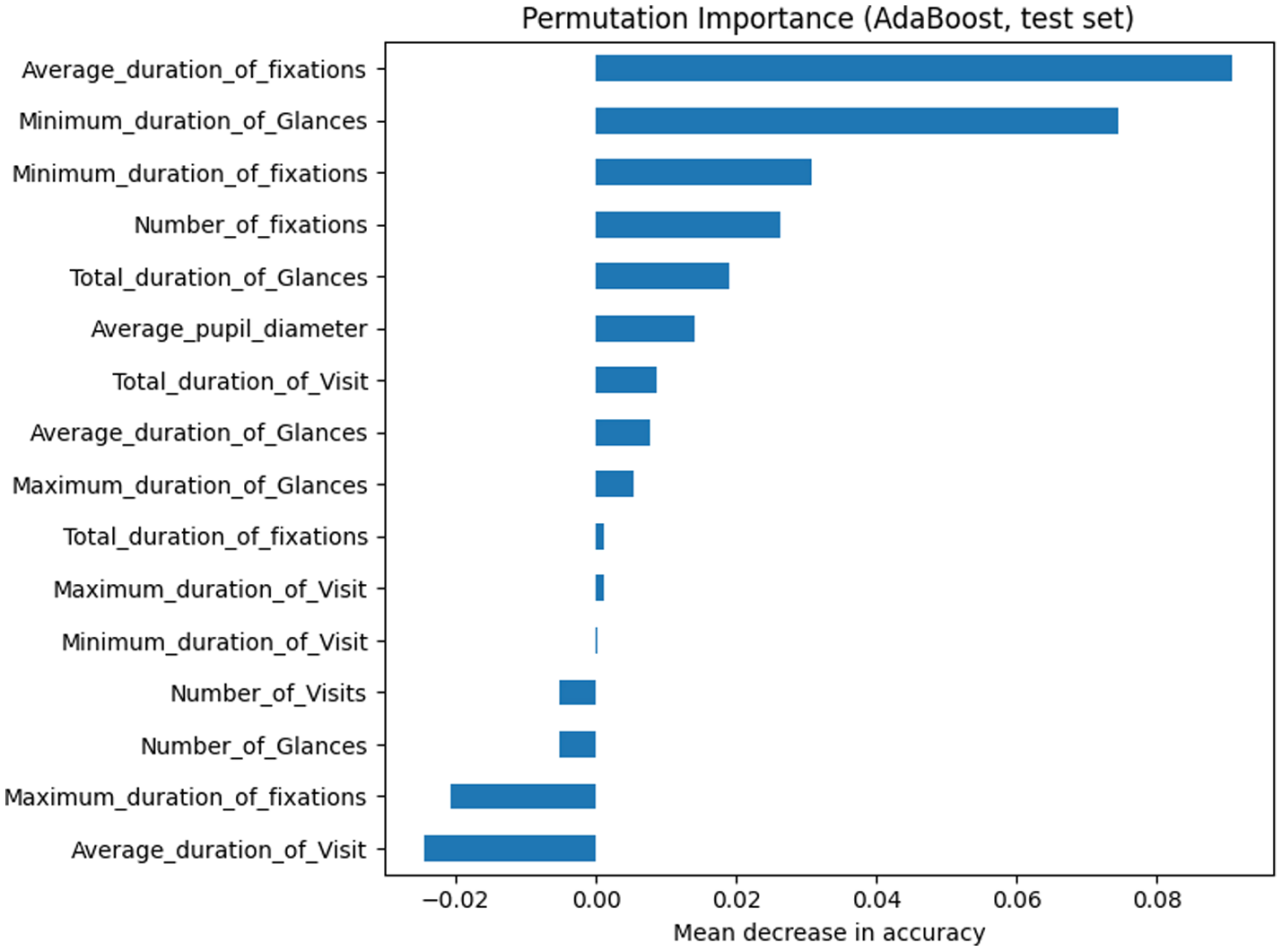
Permutation importance of gaze-based features for the AdaBoost classifier on the test set. The plot shows the mean decrease in classification accuracy resulting from random permutation of each feature, with higher values indicating greater contribution to model performance.

The permutation importance analysis conducted on the AdaBoost model revealed that temporal fixation and glance metrics were the most influential predictors of classification performance (Figure 8).

Average duration of fixations emerged as the dominant feature, indicating that fixations were strongly associated with the model’s ability to discriminate between the eye-movement patterns across the questionnaire conditions. The minimum duration of glances and minimum duration of fixations followed in importance, highlighting that even the briefest visual episodes carry discriminative information, possibly reflecting task switching and attentional micro-shifts during the reading of items. The number of fixations and the total duration of glances also contributed substantially, reinforcing the role of visual scanning intensity in distinguishing cognitive states during item response.

Among the remaining predictors, average pupil diameter showed moderate relevance, consistent with previous findings that link pupillary dilation to cognitive load and mental effort (e.g., Chen et al., 2011). Features related to total or maximum visit durations, as well as the number of visits and glances, displayed minimal contribution, suggesting that global temporal engagement measures were less sensitive for this classification task than fine-grained fixation-based features.

Overall, the explainability results indicate that fixation duration metrics are the most informative indicators of cognitive processing during questionnaire completion.

## 4 Discussion

The present study examined how visual behavior unfolds during the completion of personality questionnaires of different lengths, with the aim of identifying whether response processes vary as a function of time**-**on**-**task and instrument characteristics. Consistent with our initial framework, the results highlight a clear distinction between general temporal dynamics of engagement and instrument**-**specific patterns emerging over time.

Across both instruments, the most robust finding was a systematic temporal modulation of gaze behavior. Fixation durations, visit metrics, glance durations, and pupil diameter all varied across the three segments, typically decreasing from the initial to the middle phase and then stabilizing toward the end. This pattern suggests that time, on, task effects represent a general property of questionnaire responding, rather than a feature specific to any single instrument. Such changes are consistent with processes of habituation, increasing familiarity with the response format, or progressive optimization of response strategies. Importantly, these effects emerged consistently across all metrics, indicating that visual engagement is dynamically reconfigured as participants proceed through the task.

In line with expectations, univariate analyses revealed minimal main effects of questionnaire type, indicating that the shorter BFI-44 and the longer BFQ-2 elicited comparable overall levels of engagement when averaged across time. However, critical differences emerged when considering temporal trajectories.

Significant Test × Time interactions, particularly for fixation-based metrics (average and minimum fixation duration), showed that the two instruments diverged in how gaze behavior evolved over the course of completion. Specifically, the BFQ-2 exhibited more pronounced temporal changes, whereas the BFI-44 showed more stable patterns.

This dissociation is theoretically relevant. It suggests that response burden is not reflected in global engagement levels, but rather in how engagement changes over time. Longer questionnaires may not immediately increase effort, but they appear to create conditions under which attentional strategies progressively shift, likely reflecting adaptation, fatigue, or strategic simplification.

Eyetracking research on cognitive workload offers a nuanced interpretation of these trends. Chen et al. (2011) reported that fixation duration and fixation rate are sensitive to task complexity, with higher load associated with longer fixations and a reduced fixation rate, indicating the need for more intensive information processing. In our study, which extends the results from Casella et al. (2026), fixation duration indices emerged as the most informative features for distinguishing between questionnaires in the machine learning analyses, aligning with the view that fixation duration captures shifts in processing demands; however, the temporal decline in these measures across sections suggests that, in repetitive self-report contexts, shorter fixations may index reduced engagement and increasing satisficing. This interpretation is consistent with findings by Borys and Plechawska Wójcik (2017), who showed that more frequent refixations on the same elements were associated with poorer cognitive performance, indicating less efficient visual strategies.

While inferential analyses primarily captured temporal dynamics, the machine learning results demonstrated that gaze behavior contains additional, distributed information that is not fully captured by univariate tests.

The AdaBoost classifier achieved 73% accuracy in distinguishing between questionnaires using only gaze features from the final segment. This indicates that the overall configuration of oculomotor behavior, rather than any single metric, encodes meaningful differences between instruments.

Importantly, permutation importance highlighted fixation, based features as the most informative predictors, converging with the inferential results. This convergence strengthens the interpretation that fixation dynamics represent a key mechanism through which changes in engagement and processing strategies manifest. Taken together, these findings support the idea that response burden is a multivariate and emergent phenomenon, best captured by integrating multiple behavioral indicators rather than relying on isolated metrics.

These results have direct implications for the measurement of psychological constructs. First, they suggest that questionnaire length primarily affects the stability of engagement rather than its absolute level. Longer instruments, such as the BFQ-2, may introduce greater heterogeneity in attentional allocation across items, potentially affecting response consistency in later sections.

Conversely, shorter instruments appear to promote more stable engagement profiles, which may help preserve measurement precision. This finding is particularly relevant in the ongoing trade, off between brevity and psychometric richness, suggesting that length-related effects should be evaluated not only in terms of reliability and validity, but also in terms of response process stability.

More broadly, the study highlights the value of eye-tracking as an objective tool for assessing respondent engagement. Gaze-based measures provide continuous, fine, grained indices of cognitive effort that complement traditional self-report data, and may be especially informative in populations characterized by fluctuating attention or reduced compliance.

Several limitations should be considered. First, an important theoretical question concerns the interpretation of the observed temporal reductions in fixation-related metrics. These changes may reflect either a decrease in cognitive demand (due to increased familiarity with the task) or the adoption of more efficient or heuristic processing strategies. The absence of differential pupil-dilation patterns between questionnaires complicates this interpretation, as pupil size is typically more directly associated with cognitive load. This suggests that the observed effects may be driven more by strategic adaptation than by pure cognitive fatigue. Future studies combining eye-tracking with additional physiological measures will be necessary to disentangle these mechanisms.

Second, the sample consisted of a relatively small and homogeneous group of psychology students, limiting generalizability to broader populations. Third, because personality questionnaires were used, gaze patterns may partly reflect responses to trait, relevant content rather than questionnaire length per se.

Ultimately, the analysis focused on AOIs defined on response categories, thus capturing response selection processes but not item reading or comprehension. Finally, the single-page digital layout, while ecologically valid, may have induced specific navigation strategies (e.g., scrolling or re-scanning) that differ from other administration formats.

## 5 Conclusion

The present work examined how visual behavior evolves during the completion of personality questionnaires of different lengths, integrating inferential analyses with machine-learning methods to capture both localized and multivariate patterns in gaze behavior. Overall, the results indicate that while short and long instruments produce broadly comparable levels of visual engagement, longer questionnaires are associated with more pronounced temporal changes across fixations, visits, and glances. These findings support the idea that questionnaire length can shape the unfolding of the response process, influencing how participants visually navigate items and response options over time.

The multivariate classification results further demonstrate that gaze behavior contains sufficient structure to discriminate between questionnaires, even when individual metrics do not show strong main effects. This suggests that eye-tracking can capture subtle interaction patterns among fixation, glance-, and pupil-based measures that reflect the respondent’s evolving engagement. Importantly, these features, particularly fixation-based metrics, proved especially informative in predicting questionnaire type, underscoring their relevance for understanding how participants approach self-report items.

Beyond these empirical contributions, the findings highlight the potential role of eye-tracking in questionnaire development. Because gaze measures provide fine-grained and continuous information on how respondents interact with items, they may represent a valuable resource in early phases of validation, helping identify items that elicit atypical visual behavior, increased decision effort, or inconsistent navigation across response options. Such interaction-level insights may complement traditional psychometric analyses by revealing aspects of the response process that are not accessible through self-report alone.

Future work should expand on these results in several directions. First, analyses incorporating item text, while controlling for item length, linguistic complexity, and semantic content, would help clarify whether temporal changes in gaze behavior reflect the number of items, their content, or the interplay of both. Second, while the current study successfully distinguished between questionnaires using end-task gaze patterns, future efforts should move toward modeling the cumulative nature of response burden across the entire session. This would require the application of more complex computational models, such as Transformer-based models or Recurrent Neural Networks (RNNs), which are capable to capturing recursive temporal dependencies and the gradual evolution of cognitive states over time. Third, studies using controlled manipulations of item length and parallel forms of the same questionnaire would allow for more precise isolation of the effects attributable to instrument length. Furthermore, extending the research to larger and more heterogeneous samples will be essential for assessing the generalizability of the observed gaze patterns across different populations and personality profiles. Beyond sample size alone, age represents a critical theoretical moderator for future investigation. Developmental and aging-related factors, including shifts in attentional control, reading and response strategies, and increased fatigue susceptibility, may systematically shape oculomotor indicators of burden.

Future research should therefore employ age-stratified designs to test for age × questionnaire length interactions. Such studies would clarify how cognitive changes across the lifespan influence the visual navigation of self-report measures and whether the ocular signatures of response burden identified in the current student sample remain consistent in older or younger populations.

Lastly, future research should evaluate the robustness of these ocular indicators by comparing computerized (single-page and multi-page) versus paper-and-pencil administration. This would help determine the extent to which the observed patterns are universal or specific to the digital interface used.

Together, these efforts will contribute to a more comprehensive understanding of how respondents engage with self-report measures and how eye-tracking can inform the design, refinement, and validation of psychometric instruments.

## Data Availability

The datasets presented in this study can be found in online repositories. The names of the repository/repositories and accession number(s) can be found below: https://osf.io/wv5gp/overview?view_only=2616ad1108214261a55f9ef03844cec0.
