# Supplementary material for: Integrating eye-tracking into psychometric research: computational approaches to explore response burden in questionnaire completion
Source: Front Psychol. 2026 Jun 24;17:1757566. doi: 10.3389/fpsyg.2026.1757566 (PMC13342057; doi:10.3389/fpsyg.2026.1757566)
Supplement: Supplementary file 1 [file Supplementary_file_1.pdf]

Supplementary Material

1 Supplementary Tables

Table 1. Hyperparameter spaces explored during randomized search (25 draws per model).

Model	Hyperparameter	Search Range / Distribution	Notes
Random Forest	n_estimators	100–600 (uniform integer)	Number of trees in the forest
	max_depth	2–10 (uniform integer)	Maximum depth of each tree
	min_samples_leaf	2–5 (uniform integer)	Minimum samples per leaf node
	max_features	{√, $\log_2$, None}	Number of features at each split
	bootstrap	{True, False}	Sampling with or without replacement
Gradient Boosting	n_estimators	100–400 (uniform integer)	Number of boosting stages
	learning_rate	0.01–0.2 (log-uniform)	Shrinkage factor per stage
	max_depth	2–5 (uniform integer)	Tree depth
	subsample	0.6–1.0 (uniform)	Fraction of samples per stage
Logistic Regression	C	1×10^{-3} – 1×10^{2} (log-uniform)	Inverse regularization strength

	solver	{lbfgs, liblinear}	Optimization algorithm
	class_weight	{None, balanced}	Handle class imbalance
AdaBoost	n_estimators	50–300 (uniform integer)	Number of weak learners
	learning_rate	0.01–1.0 (log-uniform)	Weight per boosting stage
	estimator__max_depth	1–4 (uniform integer)	Depth of base learners
	estimator__min_samples_leaf	1–5 (uniform integer)	Minimum samples per leaf

Table 2. Fixations descriptives

Test	Time	Measure	N	Missing	Mean	Median	SD	Minimum	Maximum
BFQ	Initial	Total duration of fixations	24	1	12878.25	12337	5694.898	3864	25998
BFQ	Middle	Total duration of fixations	24	1	7339.17	6116.5	3699.679	3181	18753
BFQ	End	Total duration of fixations	25	0	7379.44	7148	2790.116	2198	13460
BFI	Initial	Total duration of fixations	25	0	13623.12	12000	6335.781	3031	30595
BFI	Middle	Total duration of fixations	25	0	7308.12	7545	1941.245	3064	11625
BFI	End	Total duration of fixations	25	0	7414.68	7595	2494.533	2032	13057
BFQ	Initial	Average duration of fixations	24	1	417.17	405	107.324	242	644

BFQ	Middle	Average duration of fixations	24	1	373.58	363.5	81.714	190	557
BFQ	End	Average duration of fixations	25	0	288.12	289	62.906	140	434
BFI	Initial	Average duration of fixations	25	0	380.56	385	84.018	246	631
BFI	Middle	Average duration of fixations	25	0	359.24	365	60.075	255	478
BFI	End	Average duration of fixations	25	0	363.92	372	82.617	167	506
BFQ	Initial	Minimum duration of fixations	24	1	95.17	83	34.527	67	200
BFQ	Middle	Minimum duration of fixations	24	1	87.5	83	26.05	33	133
BFQ	End	Minimum duration of fixations	25	0	46.08	46	33.309	1	117
BFI	Initial	Minimum duration of fixations	25	0	84.8	67	27.917	50	183
BFI	Middle	Minimum duration of fixations	25	0	86.64	83	21.981	67	150
BFI	End	Minimum duration of fixations	25	0	87.4	83	20.514	67	133
BFQ	Initial	Maximum duration of fixations	24	1	1693.92	1390.5	924.374	733	4430
BFQ	Middle	Maximum duration of fixations	24	1	973.54	891	263.803	566	1582
BFQ	End	Maximum duration of fixations	25	0	1036.32	933	420.226	416	2581
BFI	Initial	Maximum duration of fixations	25	0	1302.36	1216	456.013	466	2581
BFI	Middle	Maximum duration of fixations	25	0	1099.16	999	434.623	583	2548
BFI	End	Maximum duration of fixations	25	0	1035.88	949	326.614	466	1715

BFQ	Initial	Number of fixations	24	1	32.46	29	17.305	9	93
BFQ	Middle	Number of fixations	24	1	19.38	17.5	7.371	10	38
BFQ	End	Number of fixations	25	0	25.88	26	9.435	13	53
BFI	Initial	Number of fixations	25	0	35.84	32	14.418	10	73
BFI	Middle	Number of fixations	25	0	20.52	21	5.253	11	32
BFI	End	Number of fixations	25	0	20.68	19	6.362	9	30

Table 3. Pupil diameter

Test	Time	Measure	N	Missing	Mean	Median	SD	Minimum	Maximum
BFQ	Initial	Average pupil diameter	24	1	2.85	2.85	0.301	2.34	3.64
BFQ	Middle	Average pupil diameter	24	1	2.73	2.72	0.271	2.27	3.28
BFQ	End	Average pupil diameter	25	0	2.69	2.67	0.277	2.19	3.11
BFI	Initial	Average pupil diameter	25	0	2.88	2.9	0.339	2.23	3.55
BFI	Middle	Average pupil diameter	25	0	2.76	2.8	0.293	2.14	3.3
BFI	End	Average pupil diameter	25	0	2.72	2.78	0.281	2.09	3.19

Table 4. Visit descriptives

Test	Time	Measure	N	Missing	Mean	Median	SD	Minimum	Maximum
BFQ	Initial	Total duration of Visit	24	1	13856.63	13269.5	6206.243	3931	27547
BFQ	Middle	Total duration of Visit	24	1	7927.58	6753.5	3944.716	3264	20319

BFQ	End	Total duration of Visit	25	0	8015.68	8012	3037.028	2764	14083
BFI	Initial	Total duration of Visit	25	0	15032.72	13599	7240.166	3198	33942
BFI	Middle	Total duration of Visit	25	0	8004.96	8194	2039.711	3331	12158
BFI	End	Total duration of Visit	25	0	8015.48	8078	2631.116	2115	14223
BFQ	Initial	Average duration of Visit	24	1	1159.88	1121	363.51	523	2009
BFQ	Middle	Average duration of Visit	24	1	947.08	965.5	275.715	498	1432
BFQ	End	Average duration of Visit	25	0	705.44	614	479.198	251	2817
BFI	Initial	Average duration of Visit	25	0	1202.24	1133	475.242	608	2214
BFI	Middle	Average duration of Visit	25	0	977.56	910	252.709	569	1527
BFI	End	Average duration of Visit	25	0	798.04	787	219.534	353	1217
BFQ	Initial	Minimum duration of Visit	24	1	140.21	117	137.228	67	733
BFQ	Middle	Minimum duration of Visit	24	1	283.83	158.5	264.873	67	883
BFQ	End	Minimum duration of Visit	25	0	67.32	51	52.593	1	165
BFI	Initial	Minimum duration of Visit	25	0	181.92	133	159.64	50	799
BFI	Middle	Minimum duration of Visit	25	0	178.6	133	149.947	67	666
BFI	End	Minimum duration of Visit	25	0	155.32	133	91.392	67	516
BFQ	Initial	Maximum duration of Visit	24	1	3196	3143.5	1499.376	949	6395
BFQ	Middle	Maximum duration of Visit	24	1	1909.46	1690.5	829.431	883	4064

BFQ	End	Maximum duration of Visit	25	0	2389.32	1949	1931.903	916	11109
BFI	Initial	Maximum duration of Visit	25	0	3318.84	3031	1498.976	1449	8294
BFI	Middle	Maximum duration of Visit	25	0	2359.04	2565	706.625	1133	3581
BFI	End	Maximum duration of Visit	25	0	1870.6	1999	568.284	616	2815
BFQ	Initial	Number of Visits	24	1	11.92	11	4.313	6	23
BFQ	Middle	Number of Visits	24	1	8.46	8.5	3.12	4	16
BFQ	End	Number of Visits	25	0	12.32	12	3.051	5	18
BFI	Initial	Number of Visits	25	0	13	11	5.583	5	29
BFI	Middle	Number of Visits	25	0	8.52	8	2.568	4	14
BFI	End	Number of Visits	25	0	10.16	10	2.656	6	15

Table 5. Glances descriptives

Test	Time	Measure	N	Missing	Mean	Median	SD	Minimum	Maximum
BFQ	Initial	Total duration of Glances	24	1	14276.54	13677.5	6318.2	4097	28030
BFQ	Middle	Total duration of Glances	24	1	8205.21	7103	4031.19	3431	20918
BFQ	End	Total duration of Glances	25	0	8284.28	8414	3092.03	2964	14493
BFI	Initial	Total duration of Glances	25	0	15515.72	14048	7376.07	3348	35108
BFI	Middle	Total duration of Glances	25	0	8303.36	8444	2085.25	3464	12624

BFI	End	Total duration of Glances	25	0	8372	8394	2695.43	2382	14673
BFQ	Initial	Average duration of Glances	24	1	1195.08	1161	364.1	568	2047
BFQ	Middle	Average duration of Glances	24	1	980.04	999.5	276.19	535	1458
BFQ	End	Average duration of Glances	25	0	727.04	625	480.67	269	2840
BFI	Initial	Average duration of Glances	25	0	1239.16	1171	476.6	650	2254
BFI	Middle	Average duration of Glances	25	0	1012.96	958	252.85	597	1561
BFI	End	Average duration of Glances	25	0	833.8	822	221.76	397	1262
BFQ	Initial	Minimum duration of Glances	24	1	173.67	150	142.54	67	783
BFQ	Middle	Minimum duration of Glances	24	1	312.33	191.5	270.51	67	916
BFQ	End	Minimum duration of Glances	25	0	75.84	53	57.16	2	171
BFI	Initial	Minimum duration of Glances	25	0	213.88	183	166.43	67	849
BFI	Middle	Minimum duration of Glances	25	0	207.24	167	155.03	83	700
BFI	End	Minimum duration of Glances	25	0	188.64	167	99.49	83	583
BFQ	Initial	Maximum duration of Glances	24	1	3227.25	3164.5	1504.64	983	6429
BFQ	Middle	Maximum duration of Glances	24	1	1948.88	1740	832.19	916	4130
BFQ	End	Maximum duration of Glances	25	0	2415.2	1982	1934.39	949	11142
BFI	Initial	Maximum duration of Glances	25	0	3348.8	3048	1501.36	1466	8344
BFI	Middle	Maximum duration of Glances	25	0	2396.8	2581	703.93	1182	3631

BFI	End	Maximum duration of Glances	25	0	1904.6	2032	562.34	650	2831
BFQ	Initial	Number of Glances	24	1	11.92	11	4.31	6	23
BFQ	Middle	Number of Glances	24	1	8.46	8.5	3.12	4	16
BFQ	End	Number of Glances	25	0	12.32	12	3.05	5	18
BFI	Initial	Number of Glances	25	0	13	11	5.58	5	29
BFI	Middle	Number of Glances	25	0	8.52	8	2.57	4	14
BFI	End	Number of Glances	25	0	10.16	10	2.66	6	15
